# Functional Mammalian Amyloids and Amyloid-Like Proteins

**DOI:** 10.3390/life10090156

**Published:** 2020-08-21

**Authors:** Maria S. Rubel, Sergey A. Fedotov, Anastasia V. Grizel, Julia V. Sopova, Oksana A. Malikova, Yury O. Chernoff, Aleksandr A. Rubel

**Affiliations:** 1 SCAMT Institute, ITMO University, 191002 St. Petersburg, Russia; rubel@scamt-itmo.ru; 2 Laboratory of Amyloid Biology, St. Petersburg State University, 199034 St. Petersburg, Russia; serg900@yandex.ru (S.A.F.); avgrizel@gmail.com (A.V.G.); sopova@hotmail.com (J.V.S.); oks_malik@mail.ru (O.A.M.); yury.chernoff@biology.gatech.edu (Y.O.C.); 3 Pavlov Institute of Physiology, Russian Academy of Sciences, 199034 St. Petersburg, Russia; 4 St. Petersburg Branch, N.I. Vavilov Institute of General Genetics, Russian Academy of Sciences, 199034 St. Petersburg, Russia; 5 Department of Genetics and Biotechnology, St. Petersburg State University, 199034 St. Petersburg, Russia; 6 School of Biological Sciences, Georgia Institute of Technology, Atlanta, GA 30332-2000, USA

**Keywords:** amyloid screening, functional amyloid, memory, peptide hormone, protein aggregation

## Abstract

Amyloids are highly ordered fibrous cross-β protein aggregates that are notorious primarily because of association with a variety of incurable human and animal diseases (termed amyloidoses), including Alzheimer’s disease (AD), Parkinson’s disease (PD), type 2 diabetes (T2D), and prion diseases. Some amyloid-associated diseases, in particular T2D and AD, are widespread and affect hundreds of millions of people all over the world. However, recently it has become evident that many amyloids, termed “functional amyloids,” are involved in various activities that are beneficial to organisms. Functional amyloids were discovered in diverse taxa, ranging from bacteria to mammals. These amyloids are involved in vital biological functions such as long-term memory, storage of peptide hormones and scaffolding melanin polymerization in animals, substrate attachment, and biofilm formation in bacteria and fungi, etc. Thus, amyloids undoubtedly are playing important roles in biological and pathological processes. This review is focused on functional amyloids in mammals and summarizes approaches used for identifying new potentially amyloidogenic proteins and domains.

## 1. Introduction

For a long time, the term “amyloid” was used to describe extracellular tissue deposits of protein fibrils with a characteristic appearance in electron microscope (EM), typical X-ray diffraction pattern, and an affinity to Congo red dye (CR) resulting in green-yellow birefringence [1]. It was thought that amyloids are primarily associated with human and animal diseases (termed “amyloidoses”). Later studies broadened the term “amyloid” and emphasized its structural characteristics so that now it is generally used for the cross β-sheet non-covalent unbranched fibrous protein polymers formed both in vivo and in vitro. In an amyloid, β strands of repeated units are placed perpendicular to the fiber axis, forming an intermolecular cross-β sheet [2]. An amyloid polymer can immobilize non-amyloid monomeric protein molecules of the same sequence, and thus grow via a process of nucleated polymerization. Due to its highly ordered structure, amyloid fibrils as well as oligomers are characterized by resistance to ionic detergents (such as sodium dodecyl sulfate (SDS) or sarcosyl) and some proteases. In addition, they demonstrate an affinity to dyes such as CR, thioflavin T (ThT), and thioflavin S (ThS). Amyloids can also be detected with some amyloid-specific antibodies [3] or aptamers [4]. To date, 37 amyloidogenic proteins that are associated with about 70 different human diseases are known [5]. The most important amyloid-associated diseases include Alzheimer’s and Parkinson’s diseases, type 2 diabetes, and transmissible spongiform encephalopathies (TSEs), or prion diseases, such as Creutzfeldt-Jakob disease [6]. Recent data indicate that amyloids are also associated with preeclampsia and some forms of cancer, although this is unclear whether amyloids cause these diseases or arise as a consequence of the disease and serve as biomarkers [7,8,9,10,11].

In addition to pathogenic amyloids, amyloids participating in a wide range of physiological functions have been identified in various organisms, from bacteria to higher eukaryotes such as vertebrates, plants, and humans. These amyloids are termed as functional amyloids [12,13,14]. For example, the CPEB proteins of *Aplysia californica*, as well as its orthologs in *Drosophila melanogaster* and *Mus musculus* (see below) form amyloid-like SDS-resistant oligomers that are involved in the maintenance of long-term memory [15,16,17]. Spider spidroins form amyloid-based insoluble silk fibrils that are stronger than steel [18]. The protein Luminidependens of the plant *Arabidopsis thaliana*, that is capable of forming amyloid-like oligomers, is involved in the regulation of flowering by temperature [19]. Notably, this protein is a chromatin remodeler [20], albeit a connection of this function to amyloidogenecity is still hypothetical [21]. Curli proteins in *Escherichia coli* are assembled on a bacterial cell surface as a part of the extracellular matrix during biofilm formation and control resistance to a variety of environmental stresses [22,23].

In yeast and filamentous fungi, amyloid- based protein polymers can be transmitted from cell to cell during a cell division or by a cytoplasm exchange and control phenotypically detectable traits [24,25,26]. These self-perpetuating amyloids provide a basis for protein-based inheritance and are termed yeast (or fungal) prions. While some of them are clearly pathogenic, others are hypothesized or (in a very few cases) shown to be associated with adaptive roles [27]. Specifically, prion [*Het-s*] of the fungus *Podospora anserina* is involved in the control of vegetative incompatibility through the destruction of a mycelium not containing the prion at the position of contact in a manner similar to programmed cell death [28,29].

Constructs based on the translation termination factor Sup35, that can convert into the prion form termed [*PSI^+^*], are frequently applied to studying amyloid properties of mammalian and human proteins (see [30] and below).

A significant fraction of proteins involved in transcriptional regulation both in yeast and mammalian cells contain sequences, enriched by Q and/or N residues that are similar to prion domains (PrDs) of yeast prions [31,32,33] and therefore termed PrD-like domains (PrDL). However, the abilities of the majority of these proteins to form an amyloid and their potential role as amyloids are not studied.

To conclude, literature data indicate that the ability to form amyloids under physiological conditions is a characteristic feature of many proteins (including a variety of human proteins) [34], that could be linked to both pathological processes and normal biological functions. This review summarizes data on functional amyloids in mammals and describes approaches for identifying new potentially amyloidogenic proteins and domains.

## 2. Functional Amyloids in Mammals

### 2.1. Peptide Hormones in Secretory Granules

Storage of hormones inside the cell can be challenging due to their chemical activity. Some protein and peptide hormones of secretory cells such as neuroendocrine cells and exocrine cells can be stored for a long time in high concentration in membrane-enclosed secretory storage granules (StGs) until an outside signal for their excretion to the extracellular space is received. The first direct evidence for the storage of hormones and pro-hormones in granules has been reported for insulin by Steiner [35]. Further investigation indicated that growth hormone (GH) [36], prolactin [37], adrenocorticotropic hormone (ACTH) [38], and parathyroid hormone [39] form storage granules as well. Maji and colleagues later showed that over 30 of pro-hormones form amyloid structures during storage [40].

The formation of the StG starts from the self-association of hormone molecules in the Golgi complex [41]. As a rule, each StG contains insoluble aggregates of one secretory protein or peptide [42]. Amyloid properties of proteins forming these aggregates have been studied by Riek lab [14,40], by employing a set of approaches such as ThT and CR binding, luminescent conjugated polyelectrolyte probes (LCP), electron microscopy, circular dichroism (CD) spectroscopy, X-ray diffraction. 42 peptide hormones associated with StGs of multiple species and organs were analyzed. It has been shown that most of them can form amyloid aggregates [40]. Aggregation of some peptide hormones and/or prohormones can be initiated spontaneously after reaching a critical concentration threshold at pH 5.5, reflecting conditions in StGs. However, the presence of helper molecules such as glycosaminoglycans, GAGs (for example, heparin), was required for most peptide hormones, presumably for stabilizing the amyloid conformation [40]. Prolactin did not form aggregates in the presence of heparin but showed an aggregation in the presence of chondroitin sulfate A [40], which is a GAG compound found in prolactin-specific granules [43]. An aggregation of ACTH required the presence of an amyloid form of β-endorphin. ACTH and β-endorphin are processed from the same prohormone (proopiomelanocortin) and are stored in StGs together. Besides, aggregation of peptide hormones was also affected by bivalent metal cations [44]. In particular, Zn(II) was shown to initiate an early oligomerization of GH in the pituitary of rat, which may facilitate GH aggregation and amyloid formation [45].

Studies of StGs of the mouse pituitary tumor neuroendocrine cell line AtT20 confirmed their amyloid nature. Purified granules from AtT20 cells reacted to amyloid-specific OC antibodies, and were stained with ThT and CR dyes (also showing the birefringence in the latter case) [45]. In addition, X-ray diffraction of the membrane-less secretory StGs pattern was typical for the cross-β structure. Moreover, β-endorphin, GH, oxytocin, prolactin, vasopressin, and ACTH were stained by ThS or reacted to fibril-specific antibody OC in the mouse pituitary tissue [40]. It was also shown that amyloid fibrils formed by hormones can release monomers [46] if pH is increased to 7.4, the same pH to which hormones are exposed during secretion.

Some mammalian amyloids, including aggregates of the Aβ peptide (associated with Alzheimer’s disease), are toxic to neuronal cells [47]. Maji et al. demonstrated that typically, amyloid aggregates of peptide hormones formed in vitro were less toxic to culture neuronal cells than Aβ aggregates, although some aggregated hormones (for example, ovine CRH, mUcnIII, hUcnIII, human GRF, and glucagon-like peptide-2), demonstrated toxicity comparable to Aβ, whereas aggregated glucagon was much more toxic than Aβ in 3-(4,5-dimethylthiazol-2-yl)-2,5-diphenyltetrazolium bromide (MTT) assay [40]. Apparently, for these hormones condensation in StGs serves as a tool counteracting the toxicity of aggregated hormones to the cell.

Taken together, in vitro and in vivo data confirm the amyloid nature of protein and peptide hormones stored in StGs. Due to the high stability of amyloids, StGs can exist for long periods until the cell receives a signal to excrete hormones, leading to the disaggregation of amyloid assemblies into a functional soluble hormone, that is promoted by an increase in pH from 5.5 (dormant StGs) to 7.4 (blood). In addition, extracellular chaperones may be involved in disaggregation [14,40].

### 2.2. PMEL Protein and Melanin Biosynthesis

Melanins are chemically heterogeneous pigment molecules found in most organisms. There are three major types of melanin in mammals: eumelanin, neuromelanin, and pheomelanin. Eumelanin and pheomelanin are the ubiquitous pigments present in skin, eye, and hairs that effectively absorb ultraviolet (UV) and visible light [48,49] and protect against sunlight, UV radiation, small toxic molecules as well as involved in thermoregulation and control of coloring. Neuromelanin is present in the human central nervous system [50,51,52], specifically, it is found in large quantities in neurons of the *Substantia nigra*, ventral tegmental area, and *Locus coeruleus* [53,54,55]. It has been linked to etiology and pathogenesis of Parkinson’s disease [56,57]. Neuromelanin is composed of lipid, melanine, and peptide components, with the melanic portion being a mixture of pheomelanin and eumelanin [58,59].

Melanin’s synthesis takes place in melanosomes, specialized acidic organelles of pigmented cells (melanocytes). Melanosomes are derived from lysosomes. Formation of luminal amyloid fibrils originated from the melanocyte-specific pre-melanosomal protein PMEL17 has been detected during melanosome biogenesis and maturation, confirmed by ThS and CR staining of melanosomes [60]. Amyloid matrix of PMEL fibrils apparently serves as a scaffold for melanin polymerization, thus promoting melanin synthesis; it is also implicated in sequestering the toxic intermediates of melanin biosynthetic pathway [60,61,62]. These toxic intermediates possess some structural similarity to the amyloid-binding dye ThT. Binding to PMEL17 accelerates the conversion of intermediate 5,6-dihydroxyindole into the covalent melanin polymers [60]. PMEL17-derived amyloid fibrils are mainly detected during the eumelanin production, while PMEL17 participation in the formation of neuromelanin and pheomelanin remains questionable, as PMEL17 fibrils were not detected in pheomelanosomes [62,63]. PMEL17 deficiency did not affect production of pheomelanin [64] and PMEL17 has not been found at significant levels in neuromelanin-producing organelles [65]. However isolated neuromelanin shows a typical cross-β sheet structure X-ray diffraction pattern of 4.7 Å, indicating the presence of amyloid fibrils [66]. The neuromelanin-producing organelles contain alpha-synuclein and a PMEL homolog, the glycoprotein NMB, that can produce amyloid fibrils, which probably starts neuromelanin synthesis [65].

Eumelanosome maturation includes four different stages, and it is accompanied by functional and morphological changes [67]. Stage I and stage II melanosomes are referred to as immature/premelanosomal compartments, and lack pigment. Melanosome maturation is accompanied by an increase in intraluminal pH, from pH 4 in stages I and II to near neutral pH upon full maturation [68]. PMEL17 fibrils start to form in stages I and II, and are organized into parallel sheets that elongate the compartment. An acidic environment of organelles at I and II stages is optimal for the assembly of PMEL17 amyloid fibrils. However, melanin biosynthesis is suppressed at low pH, therefore it begins at stage III melanosomes and is complete at stage IV [63,69].

PMEL is a melanocyte-specific type I transmembrane glycoprotein. PMEL contains a short cytoplasmic C-terminal domain, a transmembrane domain, a luminal N-terminal domain, and an N-terminal signal peptide. The long luminal domain consists of four sub-domains: (1) N-terminal region (NTR), that contains 3 highly conserved N-glycosylation sites and 3 cysteine residues that might form disulfide bonds; (2) polycystic kidney disease-like domain (PKD), that, as predicted, might adopt β-sheet conformation [70]; (3) repeat domain (RPT) containing one conserved cysteine residues and 10 imperfect direct repeats of 13 amino acids sequence rich in glutamic acid, proline, serine, and threonine; (4) Kringle-like domain (KLD), a cysteine-rich region that also has an N-linked glycosylation site essential for protein folding and secretion [71].

**Figure 1:**
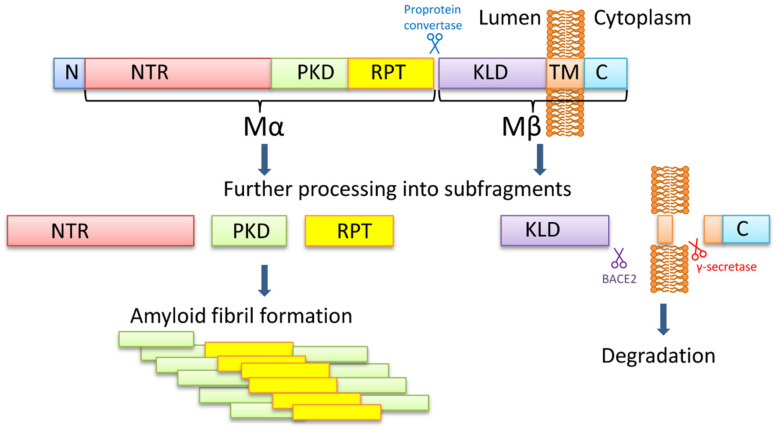
The PMEL17 processing and formation of amyloid fibrils. The PMEL17 is cut in endoplasmic reticulum and then transported to Golgi apparatus for O-glycosylation. Later in the acidic environment of premelanosomes, the proprotein convertase cuts it to the Mα (an N-terminal ectodomain) and the Mβ (the C-terminal polypeptide containing the transmembrane domain) fragments. The fragments remain connected via the disulfide bond. The BACE2 cuts the Mβ fragment out of the membrane, and so the Mα with the attached KLD domain becomes luminal. Serial cleavages later process the Mα fragment into subfragments, which can create amyloid fibrils in premelanosome. NTR—N-terminal region; PKD—polycystic kidney disease domain; RPT—proline, serine, threonine-rich repeat domain; KLD—kringle-like domain; TM—transmembrane domain; C—cytoplasmic domain.

PMEL17 fibril formation is tightly regulated and restricted to melanosomes, that allows the protection of the cell from the potential toxic effect of an amyloid (Figure 1). During synthesis, PMEL17 protein is targeted to the endoplasmic reticulum (ER), and is modified via the removal of the signal peptide, the addition of N-linked core oligosaccharides, and formation of disulfide bonds [72,73,74]. Then PMEL17 is transferred from ER to the Golgi complex, where it is O-glycosylated [75,76,77,78] and transported to the premelanosomal vesicles [68,79]. Within the acidic premelanosomal compartment, PMEL17 is proteolytically cleaved by a proprotein convertase, resulting in the large luminal α fragment (Mα), encompassing residues 25‒467 that cover NTR, PKD, and RPT sub-domains, and smaller integral membrane β fragment (Mβ), encompassing residues 468‒668 that cover the KLD sub-domain as well as transmembrane and cytosolic domains. Mα and Mβ fragments remain linked by disulfide bonds [80,81]. The Mβ transmembrane fragment is then cleaved by protease β-secretase 2 (BACE2) that releases the Mα fragment, associated with a luminal part of Mβ (MβN), from the membrane [82,83,84]. Then a series of proteolytic cleavages of Mα produce smaller N-terminal MαN and C-terminal MαC fragments, forming amyloid fibrils in acidic pH [85]. MαN contains the NTR and the PKD sub-domains, and the subsequent truncation at NTR leads to the formation of the PKD-containing fragment [86]. MαC is also further processed, resulting in a ladder of fragments [85,87].

It is still being debated which PMEL17 region is a primary amyloid-forming region. The RPT sub-domain had initially been proposed to form the amyloid core in vivo [85,87], and this has indeed been shown that RPT fragment forms amyloid in vitro at acidic pH, resembling conditions at early stages of the melanosome biogenesis [87,88,89]. Moreover, preformed fibrils can be rapidly dissolved at neutral pH, which has been proposed as an evolutionary mechanism designed to prevent toxicity of PMEL17 fibrils occasionally released into cytosol [87]. However, other researchers showed that the RPT domain is dispensable for the amyloid formation in vivo, and point to the PKD domain (residues 201–314) [86] and/or core amyloid fragment (residues 148–223) [90] as potential amyloid-forming regions, considering the RPT subdomain as a regulator of the matrix morphology, that maximizes the surface area available for pigment [91]. Further work is needed to determine the which PMEL17 region plays a primary role in the amyloid formation in vivo, although it is possible that different regions of PMEL17 possess amyloid properties, that are involved in various aspects of melanosome biogenesis and melanin production.

### 2.3. CPEB3 Protein and Long-Term Memory

Cytoplasmic polyadenylation element-binding (CPEB) protein has been implicated as a regulator of local protein synthesis at active synapses in neurons of the mollusk *Aplysia* [92]. CPEB modulates translation of mRNAs by regulating cytosolic mRNA polyadenylation (see [93,94] for review). *Aplysia* CPEB [15] and the CPEB isoform Orb2 [16] in the fruit fly *Drosophila* are shown to be required for the maintenance, but not for the formation, long-term memory and synaptic plasticity [95,96]. Notably, *Aplysia* CPEB protein exhibits prion-like properties when expressed in yeast cells [97] and can form amyloid-like oligomers, assembled into punctate structures in active synapses [15].

Human and mice genomes contain four CPEB coding genes (CPEB1-4). All mammalian CPEB protein isoforms contain RNA-binding domains at the C-terminus, but only CPEB2 and CPEB3 contain a glutamine (Q)-rich domain at the N-terminus [98]. Aggregation properties and regulation of mouse CPEB3 have been studied in more detail. Similar to some other amyloidogenic proteins CPEB3 can exist either as soluble monomer form or as a self-sustained oligomer or aggregate. Recombinant CPEB3 isolated from bacteria forms fibrils that bind CR and exhibit a green/yellow birefringence in polarized light [99]. Expression of recombinant CPEB3 fused to yellow fluorescent protein or 3 x hemagglutinin tag in yeast cells results in the formation of detergent-resistant aggregates [99], controlled by the Q-rich domain of CPEB3 and depending on the chaperone Hsp104, required for the propagation of endogenous yeast prions (see [26] for review).

CPEB3 has been shown to bind specific neuronal mRNAs, such as mRNAs for GluR2 and beta-actin, and inhibit their translation [99,100]. Monoubiquitination of CPEB3 with Neuralized1 ubiquitin ligase (Neurl1) abolished CPEB3 dependent repression and activated translation [101]. The study of the CPEB3 conditional knockout (cKO) mice revealed that the CPEB3-modulated protein synthesis is necessary for the maintenance but not for the acquisition of long-term memory [17]. Likewise, CPEB3 cKO does not affect the early phase of long-term potentiation (E-LTP) but inhibits the formation of the late phase of long-term potentiation (L-LTP) that lasts more than 24 h and is dependent on protein synthesis. The most interesting property of CPEB3 is its ability to transition from the soluble to aggregated form in response to synaptic stimulation, which leads to the cessation of target RNAs repression, and local activation of translation in active synapses [102]. Removal of the amyloidogenic Q-rich domain of CPEB3 has been shown to repress the stimulation-induced changes in the CPEB3 activity; this repression has led to profound deficits in LTP and memory [17]. Taken together, these data indicate that CPEB3 dependent upregulation of translation (supposedly due to CPEB3 conformational changes in response to neuronal activity) is crucial for the retention of memories [102].

In its soluble form, CPEB3 is SUMOylated and acts as a translation inhibitor in cytoplasmic P-bodies, non-membrane cell compartments accumulating translationally repressed mRNAs and promoting their degradation [103,104,105]. SUMOylation of CPEB3 in hippocampal neurons is decreased in response to the stimulation of learning and to the dendritic activity, accompanied by an increase in the levels of Neurl1, which leads to ubiquitination of CPEB3 and its translocation to polysomes. Translational upregulation coincides with the appearance of detergent-resistant CPEB3 aggregates [101,103,104]. As a result, protein synthesis is induced and LTP is maintained for several days [17,99]. Interestingly, SUMO-2 mRNA is also found among mRNAs upregulated in these conditions, which suggests the existence of a negative feedback loop including CPEB3 and SUMO proteins, a possible regulatory mechanism preventing excessive aggregation of CPEB3 [103].

According to the model proposed by Si and Kandel [102], the CPEB-dependent activation of translation in active synapses is mediated by an aggregated form of CPEB, as aggregates contain the exposed RNA binding domain [102]. Binding of multiple mRNAs on the surface of the fibrous CPEB3 scaffold could allow for the coordinated translation of a variety of coregulated mRNAs, products of which are required for the stabilization of synaptic growth. It is however unclear whether self-sustaining fibrillar amyloids or small oligomers are crucial for translational activation. Existing data are also compatible with an alternative hypothesis, suggesting that aggregation of CPEB induces translation via downregulating the repressive activity, exhibited by the soluble form of CPEB [106]. Besides, it has been reported that the CPEB-dependent activation of translation in *Aplysia* is required only during the first 72 h after training; after that period, protein synthesis inhibitors and CPEB knockdowns no longer affect synaptic growth [107]. Thus, further studies are needed to decipher the exact mechanism by which CBEP3 oligomerization and/or aggregation modulate long-term potentiation and memory.

A recent study of Hervas and colleagues confirmed an amyloid structure, revealed a formation of the amyloid core, and thoroughly described a mechanism of activity [108]. According to the group, the Orb2 filament was composed of a hydrophilic core with a stabilization via interdigitated glutamines [108].

### 2.4. RNA-Binding Protein FXR1

Fragile X-related Proteins (FXR1 and FXR2) are RNA-binding proteins that regulate transcription, translation, and RNA stability [109,110,111]. FXRs are associated with ribosomes, predominantly with the large (60S) ribosomal subunits [112], and with the RNA-induced silencing complex via Argonaute 2 (AGO2) [113,114]. Small FXR1/AGO2-containing ribonucleoprotein (RNP) granules associate with TNFα AU-rich elements (ARE) facilitating reporter translation under conditions of growth inhibition. However, in dividing cells, FXR1 forms insoluble complexes and the TNFα 3′-UTR-containing reporter RNA translation is blocked [115,116]. The repression of translation is accompanied by translocation of FXR1 from the AGO2-bound polysomal mRNP to the P-body positive foci [115]. In addition, FXR1 is possibly involved in the formation of stress granules known to accumulate stalled translation initiation complexes during unfavorable conditions [117,118]. It was shown that the highly conserved N-terminus of human FXR proteins forms aggregates with amyloid properties in vitro [119].

Using PSIA-LC-MALDI approach (proteomic screening and identification of amyloids/liquid chromatography coupled with mass-spectrometry) for searching amyloids in the rat brain, Sopova et al. observed that FXR1 forms SDS-resistant aggregates of amyloid type [120]. Authors demonstrated that FXR1 is binding CR, ThS, and ThT in rat brain cells, as well as in human neuroblastoma cell culture. Formation of the amyloid form of FXR1 in tissues possibly depends on the conserved N-terminal fragment of the protein. It was suggested that an aggregated state of FXR1 is important for the inhibition of translation and protection of mRNA from degradation [120], which could be crucial for long-lived cells such as neurons.

### 2.5. Zona Pellucida Proteins

Zona Pellucida (ZP) is an extracellular fibrillar coat of oocytes that plays a vital role during fertilization and preimplantation development. ZP consists of three (mouse) or four (human) glycoproteins (ZP1–4) that are produced by growing oocytes [121,122]. Each ZP protein contains a ZP polymerization domain that controls the formation of fibrils and their assembly into a porous three-dimensional ZP matrix [123]. Mouse ZP1 and ZP2 are shown to form heterodimers with ZP3, which are assembled into long filaments cross-linking through ZP1 homodimers [124,125,126]. Isolated ZP possess amyloid features, as shown by their reaction to amyloid-specific antibodies, CR binding and green/yellow birefringence, ThS binding, and detergent resistance [127]. So, all three mice ZP proteins are probably included in the ZP matrix in an amyloid shape.

Comparison of the ZP3 polymerization domains among various vertebrates from fish to humans demonstrated the lack of overall sequence conservation; however, conserved stretches were found in sites that are predicted to be amyloidogenic [127]. Some of these sites are localized in regions for which the formation of β-strands is expected according to the crystal structure of monomeric chicken ZP3, including regions important for the interaction between ZP subdomains [128]. Other amyloidogenic sites have been found in regions located outside of the ZP polymerization domain, including ZP-N repeats, which mediate interactions with spermatozoid cells [129]. None of ZP proteins contains QN-rich prion-like domains that would be similar to aggregation-prone sequences of yeast prion proteins.

Like some other functional amyloids, ZP can switch between distinct functional states, as a part of the cell physiological response to external factors. After the sperm and egg fusion, a cascade of biochemical reactions is launched, leading to the exocytosis of cortical vesicles that release proteases and other enzymes into the perivitelline space between ZP and the cell membrane and modify ZP by cleaving the polypeptide fragment from ZP2 [130]. As a result, additional spermatozoa are no longer able to penetrate ZP. Transgenic female mice with truncated ZP2 are sterile [131]. The presence of 6–8 amyloidogenic sites in each of the ZP proteins generates a potential for a variety of cross-β structures [132].

ZP amyloids may protect the oocyte after fertilization when it is surrounded by proteases and other hydrolytic enzymes released from the sperm. The protective function of ZP amyloids is probably required in subsequent processes of early embryogenesis since ZP holds together the blastomeres devoid of cell contacts [133,134]. In addition, based on the fact that the acrosomal matrix (insoluble fraction in the acrosome of spermatozoon) has an amyloid cortex, which includes ZP binding proteins, it is suggested that sperm-ZP binding can also occur through amyloid-amyloid interactions [132].

### 2.6. RIP1 and RIP3 Proteins

RIP1 and RIP3 are critical receptor-interacting serine/threonine kinases responsible for mediating necroptosis [135,136,137], a process that has been implicated as an important driver of inflammation and pathology in certain human diseases, such as ischemic brain injury, immune system disorders, some forms of neurodegeneration, and cancer [138,139,140]. Necroptosis is one of the regulated forms of necrosis. Conventionally, necrosis was considered as unprogrammed cell death in contrast to the standard programmed cell death via apoptosis, however recent data uncovered multiple caspase-independent pathways of regulated necrosis, including RIP1/RIP3-mediated necroptosis [136,141,142]. Both RIP1 and RIP3 contain the N-proximal Ser/Thr kinase domains (KDs), encompassing aa residues 17–289 (RIP1), or 21–287 (RIP3), and RIP homotypic interaction motifs, (RHIMs) encompassing aa residues 531–547 (RIP1) or 450–466 (RIP3) [143]. RIP1 also contains a C-terminal death domain” (DD) located at aa positions 583–669 and implicated in the recruitment of RIP1 into the TNFR1 (tumor necrosis factor receptor 1) signaling complex [144,145,146]. RIP1 and RIP3 can form mixed amyloid fibrils mediated by their RHIM domains, and this amyloid-based complex actually serves as an inducer of necroptosis [137]. The amyloid nature of the RIP1/RIP3 complex was confirmed in vitro by its ability to bind ThT and CR, circular dichroism, infrared spectroscopy, X-ray diffraction pattern, and solid-state NMR analysis, as well as in vivo by using ThT staining of RIP1/RIP3-containing puncta in necrotic HeLa cells [147].

It appears that various modifications of RIP1 determine various cell fates, such as survival, apoptosis, or necroptosis. For example, ubiquitination of RIP1 promotes cell survival due to activation of nuclear factor kappa B (NF-κB) and mitogen-activated protein kinases, while non-ubiquitinated RIP1 induces the caspase-8-mediated apoptosis pathway [148]. Amyloid RIP1/RIP3 complex, also known as necrosome or ripoptosome is formed in the conditions when caspase-8 is inhibited [136]. During necrosome formation, RIP1 and RIP3 autophosphorylate and transphosphorylate each other [136,149]. Then, RIP3 phosphorylates the mixed lineage kinase domain-like protein (MLKL) [143], resulting in its oligomerization and translocation in the membrane that causes pore formation and disruption of membrane integrity [139,150]. Interestingly, some viruses such as Herpes Virus 1 [151] or Epstein–Barr Virus [152] interfere with amyloid formation via RHIM-less proteins mimicking RIP1 and RIP3.

Some mutations in the RHIM domains of RIP1 or RIP3 impair amyloid formation and protect cells from necroptosis in vitro, thus signifying the importance of an amyloid formation for the process of necroptosis [147]. Presumably, RIP1 and RIP3 form hetero-amyloid structures. High-resolution solid-state NMR structural studies of amyloids formed by RIP1, RIP3, or RIP1 and RIP3 together show that the RIP1/RIP3 heteroamyloid is energetically favorable in comparison to either homoamyloid [153]. To date, the immunoprecipitation method revealed that RIP1/RIP3 can compose a heteroamyloid [149], although the structural and functional relationships require further clarification. It is also worth noting that the RHIM motifs of RIP proteins are homologous to the prion domain of Het-s, a prion protein of the fungus *Podospora anserina*, that is also involved in the programmed mycelium destruction process, controlling the cytoplasmic incompatibility, a biologically advantageous process in fungi [154,155]. Proteins with similar domains and roles are also found in other fungi [156]. These findings point to the evolutionary conservation of the amyloid-based triggering of programmed cell death.

### 2.7. MBP-1 Protein

Eosinophil major basic protein 1 (MBP-1) is a component of eosinophil membrane-enclosed granules that are released in the inflammation foci. These granules also include two ribonucleases, ECP and EDN/RNase2, and peroxidase EPO, which together participate in the destruction of bacteria, viruses, and helminths [157,158]. Inactive MBP-1 is accumulated in granules in the form of amyloid-type aggregates that could be stained with ThT and conformation-specific antibodies [159]. The MBP-1 monomers aggregate in vitro, as visible by transmission electron microscopy [159].

When eosinophils are activated at the site of inflammation, granules are acidified, and their contents are released into the extracellular space by piecemeal degranulation [157,160]. Acidification of the granule contents promotes disassembly of insoluble aggregates into monomers and/or oligomers, and therefore, conversion of MBP-1 into an active toxic form [159]. This secretion into the neutral pH of cellular milieu favors binding of soluble MBP-1 to the surface of bacterial cells, resulting in the membrane damage and bacterial death. It is believed that after binding to the bacterial membrane, activated MBP-1 aggregates into toxic β-sheet-rich clusters.

In silico analysis reveals a 5-aa stretch in MBP-1 that shows a tendency to form β-crosslinks [159]. It was shown that MBP-1-derived peptide encompassing aa residues 18–45 [161] and including this amyloidogenic stretch, causes toxicity via thinning the keratinocyte layer and promoting DNA fragmentation in keratinocytes when injected into the mouse dermis [159].

Several diseases associated with massive eosinophil infiltration and degranulation, such as eosinophilic asthma, atopic dermatitis, etc., are caused by MBP-1 toxicity to the host cells in inflammatory loci [162,163]. Anti-amyloid antibodies and other amyloid-binding substances such as heparin accelerate MBP-1 aggregation and significantly suppress the toxic effect of MBP-1 [164]. In addition, large extracellular amyloid deposits of MBP-1 are often found both in tissues infiltrated with eosinophils and in those with relatively little evidence for eosinophil-associated tissue damage [165,166]. Heparin is known to be released by mast cells, granulated white blood cells, which are also present in the sites of inflammation. This could serve as a mechanism accelerating aggregation and subsequent inactivation of MBP-1, and therefore protecting tissues from the toxic effects of MBP-1 [159,167].

### 2.8. CRES and PAP

Functional amyloids have also been found in the epididymis, a convoluted tubule that is located in the testis. During migration through the epididymis, spermatozoa undergo maturation and acquire motility and the potential for fertility. The maturation of spermatozoa requires interactions with proteins secreted by the epididymal epithelium. The epididymis is also involved in protection against pathogens [168]. Proteins secreted by the epididymal epithelium produce an amyloid-based matrix, consisting of multiple members of the family 2 cystatins, including the cysteine protease inhibitors (such as cystatin C) and four members of the cystatin-related epididymal spermatogenic subgroup, namely CRES, CRES2, CRES3 and cystatin E2 [169].

Several standard techniques such as binding to ThS, ThT, and anti-amyloid antibodies, X-ray diffraction, and negative-stain transmission electron microscopy confirmed the amyloid nature of the CRES-based epidydimal matrix [168]. However, the physiological function of these amyloids is not clear. In mouse models, it has been demonstrated that disruption of the amyloid matrix can result in epididymal pathology, including infertility [170,171], lysosomal storage disease, and decreased sperm survival [172].

Amyloid structures were also found in the acrosomal matrix (AM) and an equatorial segment of a spermatozoon, as confirmed in vivo by ThT staining and by resistance to SDS and formic acid treatments [132]. AM contains fifty-nine proteins, including known amyloidogenic proteins such as cystatin C, CRES (same as in epididymal matrix), lysozyme, transglutaminase 3, zonadhesin, zona pellucida 3 receptor and others [173]. To date, it is not clear which proteins form amyloid in AM. Moreover, the functional role of the amyloid structures in AM is not determined. It was suggested that the AM amyloid could be crucial for the acrosome exosomal reaction during fertilization and/or for proper orientation of signaling complexes during fertilization [129,172,174].

Prostate acidic phosphatase (PAP) is a semen-derived protein, which is not studied well yet, but there is some evidence of the formation of amyloid structure by this protein and in particular, by its region encompassing aa residues 248–286, based on X-ray scanning [175]. It tends to form structured fibrils in the alkaline or neutral environment but produces amorphous agglomerates in the acidic environment [176]. PAP fibrils apparently produce a fibrous network in the vagina. Additional evidence of the amyloid nature of PAP fibrils is that they are destroyed by anti-amyloid β agents (such as (−)-Epigallocatechin-3-gallate [177]) or by yeast chaperone Hsp104 [178] involved in the fragmentation of yeast amyloid-based prions [26,179].

It is possible that the PAP network is involved in antibacterial protection during sexual intercourse [180]. Unfortunately for humankind, some viruses such as HIV or herpes virus have adapted to this mechanism, so the naturally occurred protection mechanism now provides an increase of virus infiltration 4–5 fold [181]. Locking the virus with the mechanism described above is one of the promising ways to decrease virus spreading [182].

### 2.9. MAVS Proteins

The system composed of the MDA5 (melanoma differentiation-associated 5) protein and MAVS (mitochondrial antiviral signaling protein) is involved in defense against viral agents and targets genetic material or replication intermediates of many viruses [183]. MDA5-MAVS system is a primary aim of the viral immune system. Hepatitis C [184], enterovirus 71 [185], and coxsackievirus [186] target MDA5 and inhibit downstream signaling.

MDA5 includes the N-proximal caspase recruitment domains (CARDs), middle DExD/H-box helicase domain, and C-terminal domain (CTD) [187]. MDA5 binds non-specifically to long nucleic acid molecules that are frequently associated with viral invasion (such as dsDNA viral genomes and viral replication products e.g., folded RNA) via its helicase and CTD domains and forms a polar helical structure with a twist around dsDNA [188]. This conformation allows exposure of CARDs, enabling them to bind MAVS proteins, anchored into membranes of mitochondria and peroxisomes [189]. Thus, CARDs of MDA5 seed the MAVS filaments formation. MAVS protein consists of the transmembrane domain, long cytosolic domain containing the CARD-binding site, and targeting domain (TM). Amyloid nature of MAVS fibrils was confirmed by electron microscopy, resistance to detergents (using semidenaturating detergent-agarose gel electrophoresis), and resistance to high concentrations of proteases; MAVS depositions were also studied using fluorescence microscopy [190]. Polymeric (but not monomeric) MAVS activates downstream RLR signaling pathway by inducing IRF3 and HF-kB via its TM domains [191,192]. Hou and colleagues report that one mitochondria with polymerized MAVS can initiate the same process on another MAVS protein in another mitochondria and so drastically increase signal propagation [190]. On the other hand, a cell can regulate the RLR signaling activity with a truncated alternatively spliced form of MAVS (mini MAVS) antagonizing activation by polymeric MAVS [193].

### 2.10. Fibrin

Fibrin is a polymeric protein component of blood clots, formed from fibrinogen via its cleavage by thrombine protease, followed by polymerization. Fibrin mesh is necessary for the prevention of blood loss and healing the wounds [194].

Fibrinogen is a hexamer of three pairs of polypeptide chains, Aα, Bβ, and γ [195,196]. During fibrin formation, thrombine cleaves the small fibrinopeptides A and B (FpA and FpB) of fibrinogen to yield the α, β and γ chains, forming the fibrin monomer. Then monomeric fibrin self-assembles spontaneously to yield polymeric fibrin mesh [194].

The first evidence of amyloidogenic properties of fibrin was reported by Kranenburg et al. [197], who demonstrated that in the conditions, mimicking the physiological situation, a peptide corresponding to the aa positions 148–160 within the fibrin α chain can form amyloid fibrils in vitro, as confirmed by ThT binding and X-ray diffraction patterns. The fibrin peptide and fibrin in the polymerized cross-β form are binding tissue plasminogen activator (tPa), thus stimulating tPA-mediated plasmin formation, and proteolysis of fibrin itself (fibrinolysis) in vitro [197]. Moreover, Kranenburg et al. showed that some other proteins in cross-β form (Aβ, Human islet amyloid polypeptide (hIAPP) and endostatin) also support tPA-mediated plasminogen activation [197].

However, it is still not clear whether or not fibrin forms the amyloid-type cross-β structure in vivo. Some authors suggested that polymerized fibrin retains α-helical secondary structure [198,199,200,201], but can undergo structural transformation into cross-β amyloids under certain conditions, such as mechanical stretching [202,203], in individuals with certain amyloidosis such as Alzheimer’s or Parkinson’s diseases, type 2 diabetes and others [204,205,206,207,208], or in a result of a mutation [11,209,210,211]. Thus, the α-helix to β-sheet transition could be a sign of an anomalous blood clotting, leading to hypercoagulability and hypofibrinolysis [212]. For example, Aβ (a peptide related to Alzheimer’s disease, which is also present in blood) binds fibrinogen with high affinity, and such an interaction induces abnormal fibrin clotting that is resistant to degradation [213,214,215]. These abnormal clots are stained with ThT, but it is not clear whether fibrin in clots is in amyloid form or not, and whether fibrinogen provokes aggregation of Aβ or vice versa [212]. According to these findings, amyloid formation by fibrin is mostly related to pathological amyloidosis rather than to the fibrin function. However, this issue remains unclear, and further investigation on the role of fibrin-based amyloid fibrils in vivo is needed.

### 2.11. TIA-1 Protein and Stress Granules

During stress, cells have to suppress particular metabolic pathways and block some functions, simultaneous increasing production of components needed for the defense against stress, such as heat shock proteins. For the rapid reorganization of the translational machinery, eukaryotic cells transiently assemble the non-essential translational pre-initiation complexes into formations, termed stress granules (SGs). SGs contain mRNAs (not coding for the stress response proteins), translational factors, and small ribosomal subunits, as well as some additional components, such as helicases, ribonucleases, kinases, and signaling molecules [216]. After return to normal conditions, SGs disassemble; otherwise, they can turn into solid aggregates [217].

SGs are non-membranous organelles, which possess features of liquid-liquid phase separation assemblies (biocondensates); however, they are nucleated by a protein that has been historically named T-cell intracellular antigen-1 (TIA-1) and is suspected to possess amyloid properties. TIA-1 contains RNA-recognition motifs at the N-terminus and a Q/N-rich domain, similar to prion domains (PrDs) of yeast proteins, and therefore termed the PrD-like domain (PrDL), at the C-terminus [218]. TIA-1 is associated with mRNAs via the RNA-recognition motifs, while PrDL mediates interactions between TIA-1 molecules. TIA-1 formed fibrillar structures in vitro; when expressed in yeast, mammalian TIA-1 produces filamentous detergent-resistant polymers with prion-like features and interacts with PrD of the yeast translation termination factor Sup35 [218]. Notably, Sup35 PrD can substitute for the TIA-1 PrDL domain in the process of SG nucleation in mammalian cells [219]. Additionally, Sup35 PrD fused to hemagglutinin tag is recruited into SGs in non-stressed cells of the N2a cell line [220]. Rayman and Kandel reported a transition of TIA-1 from the monomeric state into an SDS-resistant (possibly amyloid-like) structure in a mouse brain [221]. Notably, TIA-1 regulates the synthesis of stress-related chaperones Hsp40 and Hsp70 [219,222]. Still, it remains unclear whether TIA-1 polymers formed in the process of SG formation represent amyloids.

The TIA-1-deficient mice are viable, but exhibit high mortality at early stages of development and elevated susceptibility to lipopolysaccharide-associated toxicity [223], as well as alterations in lipid dynamics [224]. The depletion of the TIA-1 also increases susceptibility to a viral infection, in agreement with increased accumulation of SGs are the sites of viral assembly in wild-type cells [225]. Some viruses such as rhabdovirus modulate or inhibit SG formation [226]. There is also evidence of the association of the AD-related amyloidogenic proteins, Aβ, and tau with SGs [227]. Tau, in particular, plays an important role in the formation of solid aggregates via regulating TIA-1 distribution and promoting SG formation and transition to the solid aggregated state [228].

Information about all the proteins described in this section is summarized in Table 1.

## 3. Approaches for Identification of New Amyloids and Potentially Amyloidogenic Proteins

Most amyloids known to date were identified either due to their accumulation in specific diseases or through detailed studying of the mechanisms of the impact of specific proteins on particular biological or pathological processes. Unfortunately, the initial identification of many amyloids and potentially amyloidogenic proteins in biological samples cannot be carried out based on Congo red staining, EM or X-ray diffraction data, because these can be used for characterization of purified proteins or large deposits, but are not directly applicable to the detection of small qualities of fibrils or oligomers proteins in the cells, organisms or body fluids. Until recently, no unbiased approaches for the identification of amyloids and potentially amyloidogenic proteins existed. While the formation of insoluble aggregates is a characteristic feature of many amyloids, not all insoluble aggregates are amyloids. Various computational tools for amyloid prediction were developed, however most of them work well for short peptides and/or in vitro conditions, but not for full-length proteins in vivo (for review, see [229,230]). The matter is further complicated by the fact that many (if not most) proteins can form amyloid in vitro depending on concentrations and conditions (such as pH, etc.) [231]; however, amyloidogenic potential of the majority of protein sequences is suppressed in cells and organisms, as it interferes with normal protein functions. Therefore, there is a significant demand for developing unbiased approaches to identification of in vivo, amyloid potential, capable of composing actual and potential “amyloidomes” (i.e., complete cells of amyloid or potentially amyloidogenic proteins in proteomes) of living cells and organisms.

### 3.1. Identification of Amyloid Proteins Based on Their Biochemical Properties

**Figure 2:**
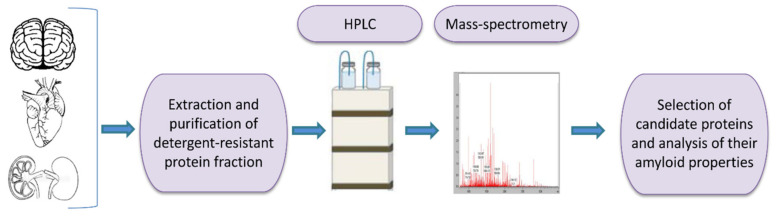
A scheme of proteomic screening and identification of amyloids (PSIA) method for amyloid screening. The pellet fraction of a homogenized sample, that is resistant to sodium dodecyl sulfate or sarcosyl is analyzed by high performance liquid chromatography followed by mass-spectrometry. Individual candidate proteins, identified by PSIA, can be further investigated in vivo and in vitro using conventional approaches such as circular dichroism spectrum, X-ray fiber diffraction, ability to bind Congo red dye with green/yellow birefringence in polarized light, etc.

One of the tools for universal identification of amyloids is based on a common biochemical property of most amyloid fibrils, that is, their high resistance to ionic detergents such as SDS or sarcosyl, which solubilize almost all non-amyloid complexes and disrupt lateral interactions between amyloid fibrils, but don’t monomerize fibrils per se [232]. Amyloids are sedimented from detergent containing solutions and further analyzed by either 2D gel electrophoresis, or liquid chromatography followed by mass-spectrometry (Figure 2) [117,233,234,235]. By using the latter combination, the proteome-wide approach termed PSIA (Proteomic Screening and Identification of Amyloids) has been developed [117,234,235]. PSIA method was applied to identification of new amyloid proteins (potential functional amyloids) in bacteria [236,237] and yeast [238], as well as in the rat hippocampus [120]. Amyloidogenic properties of some of these proteins, including bacterial proteins YghJ, RopA and RopB, yeast proteins Gas1, Toh1 and Ygp1, and the abovementioned rat Fxr1 have been then confirmed by using a variety of standard amyloid characterization techniques [117,237,238,239,240]. An approach similar to PSIA has been independently developed by F. Shewmaker lab and applied to identification of yeast prions [241].

A disadvantage of this approach is that PSIA is not completely selective and can also detect some non-amyloid formations that are stable in detergent [120]. In addition, proteins forming amyloids only in certain physiological conditions would not be detected if proper conditions are not used.

### 3.2. C-DAG

**Figure 3:**
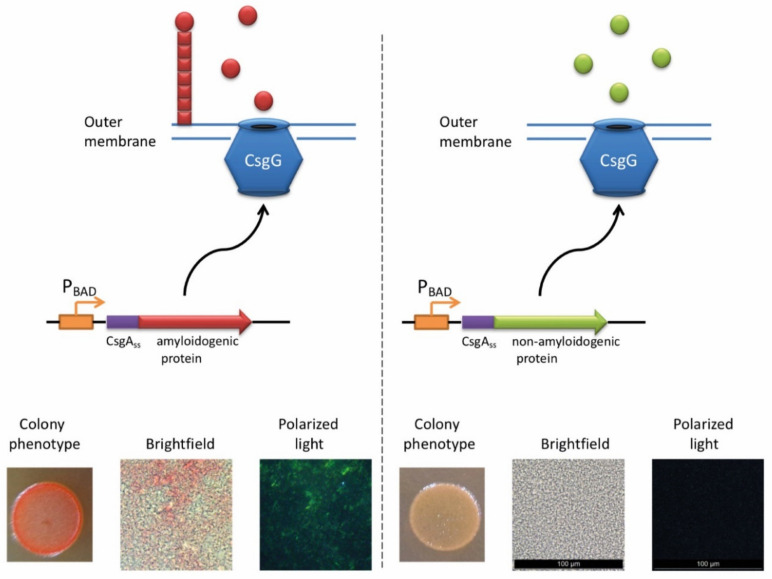
The principle of C-DAG approach (for detail, see [244]). A chimeric protein, containing the export signal sequence of CsgA protein fused to the protein of interest is exposed to the extracellular space. If the protein of interest is amyloidogenic, it forms a “curli”-like fibril. Amyloid-producing colonies become reddish on the medium containing Congo red (CR). Moreover, binding of CR to amyloid fibrils results in apple-green birefringence in polarized light. Images are obtained by J.V. Sopova (unpublished data).

The ability of *E. coli* cells to produce extracellular fibers known as “curli”, inspired the creation of a new system for amyloid detection called curli-dependent amyloid generator (C-DAG) [242]. When the export signal sequence of CsgA protein is fused to an amyloidogenic protein (or its amyloidogenic domain), the resulting chimeric protein is secreted and can form amyloid fibrils, anchored into the outer membrane and exposed to the outside environment (Figure 3). When plated on Congo red containing media, these amyloid-producing colonies become reddish due to the binding of CR to amyloids. Amyloid nature of CR-bound fibrils is further confirmed by apple-green birefringence in the polarized light, and fibrils can also be visualized by electron microscopy [243]. This approach was successfully tested using several known yeast amyloidogenic proteins (Sup35NM, Rnq1, Cyc8, and New1) and polyQ (aggregating) region of human huntingtin (Htt72Q), while the non-aggregating linker domain of yeast Sup35 protein (Sup35M) and non-aggregating derivative of human huntingtin (Htt25Q) were used as negative controls [242]. At subsequent stages, the C-DAG system was used to confirm the amyloidogenic properties of several yeast proteins (Mss11 and Pub1 [242], Gas1 and Ygp1 [238], and Toh1 [240]), some bacterial proteins (biofilm-associated proteins especially from *Enterococcus faecalis* and Bap from *S. aureus* [244], RopA and RopB from *Rhizobium leguminosarum* [237], and YghJ from *E.coli* [239]), and Fxr1 protein from rat *Rattus norvegicus* [120]. C-DAG approach was suggested as a convenient method for screening of potentially amyloidogenic proteins from DNA libraries [242]. This approach can also be used to identify mutations that influence the process of amyloidization, as shown for the Sup35NM variant lacking four oligopeptide repeat sequences [242]. However, the C-DAG approach has several limitations. First, colony coloration may vary depending on the particular protein [242]. Second, not all mammalian proteins are efficiently produced and exported in *E. coli* [245]. Third, an extracellular environment might potentially antagonize amyloid formation.

### 3.3. Approaches Based on Phenotypic Detection of Prion Formation in Yeast

The yeast *Saccharomyces cerevisiae* contains endogenous self-perpetuating amyloids (yeast prions) that serve as a convenient and reliable model for studying amyloid aggregation (for review, see [25,26]). Aggregation of a variety of mammalian human amyloidogenic proteins has also been reproduced and studied in yeast. These topics, especially in regard to studies of pathogenic mammalian amyloids in yeast, have been comprehensively covered in recent reviews (see [26,30]). Here, we only mention yeast-based approaches that can be applied to the identification of new amyloidogenic proteins (among them, potential functional amyloids).

The major advantage of the yeast model is that amyloid formation by yeast proteins can be detected phenotypically, via growth and/or color of specially engineered yeast strains on specific media. These phenotypes are usually resulting from the partial inactivation of a yeast protein in a prion form, thus chimeric constructs based on the fusion of a portion of the yeast protein to the potentially amyloidogenic region of a mammalian protein can utilize yeast protein as a reporter for the amyloid formation by a mammalian protein, allowing for easy phenotypic readout.

**Figure 4:**
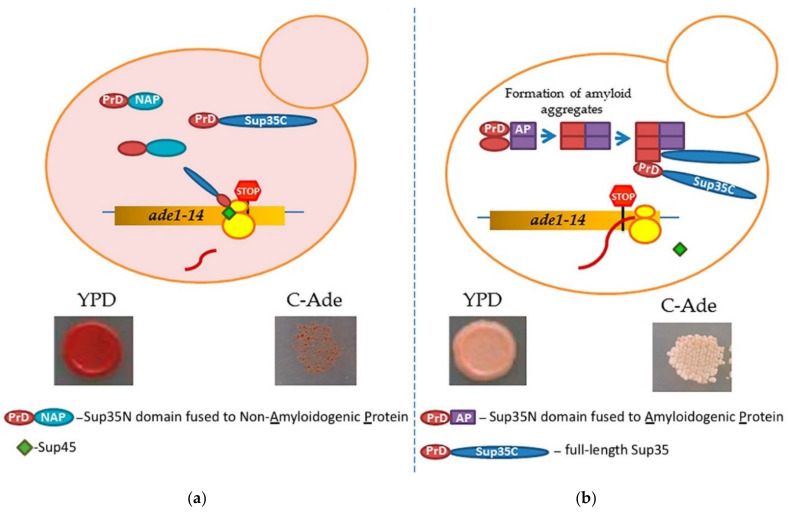
A scheme of testing heterologous proteins for amyloidogenicity in yeast. Yeast strain contains an *ade1-14* (UGA) reporter, allowing the detection of the Sup35 prion ([*PSI^+^*]) due to translational readthrough. (**a**) Overexpression of Sup35 PrD alone or in fusion to non-amyloidogenic protein (PrD-NAP) does not lead to efficient nucleation of the [*PSI*^+^] prion in the [*pin*^−^] cells yeast lacking any known pre-existing prions. Respectively, there is no growth on the medium lacking adenine, and red color is detected on complete (YPD) medium due to the accumulation and polymerization of an intermediate in the adenine biosynthetic pathway. (**b**) An amyloidogenic protein (AP) attached to Sup35 PrD forms an amyloid aggregate in yeast, thus bringing together the Sup35 PrD regions and facilitating the conversion of these regions into a cross-β nucleus. Complete Sup35 protein, present in the same cell, is then immobilized into such a nucleus via the PrD-PrD interaction, and converted into a prion form, thus producing the [*PSI^+^*] prion and allowing for a phenotypic detection. Respectively, there is growth on the medium lacking adenine, and a whitish color is detected on YPD medium.

Prion-forming protein Sup35 that is most frequently employed in such studies is a yeast counterpart of the translation termination (release) factor, eRF3 [246,247]. Sup35 protein is composed of three major regions, namely: (a) N-terminal PrD, or Sup35N that is responsible for amyloid formation, (b) middle region or Sup35M that promotes Sup35 solubility in a pH-dependent manner, and (c) the C-proximal functional region, that is essential and sufficient for the role of Sup35 in termination of translation [25,248]. While Sup35N and Sup35M are dispensable for translation termination and cell viability, Sup35 can antagonize this function of Sup35C by incorporating the Sup35 protein into [*PSI^+^*] prion aggregates. Prion aggregation of Sup35 results in a decrease of its ability to access terminating ribosomes, thus causing readthrough of nonsense codons [25]. Therefore, formation of the Sup35 prion state can be phenotypically detected, for example in yeast strains with a premature stop codon in the *ADE1* gene (the UGA mutation *ade1-14*). In normal conditions, such a strain is typically incapable of growing on the medium lacking adenine (-Ade) and accumulates red pigment (a polymerized intermediate of the adenine biosynthetic pathway) on complete organic (Yeast extract with Peptone and Dextrose, or YPD) medium. However, UGA readthrough due to partial inactivation of Sup35 in the prion ([*PSI^+^*]) form leads to growth on -Ade and more whitish color on YPD (Figure 4).

One approach for uncovering and/or studying amyloidogenic potential of proteins of various origins (for example, see [249]), is based on the substitution of the whole Sup35N domain (or in some cases, its N-terminal aggregation-prone QN-rich stretch, e.g., see [250]) by the known or suspected amyloidogenic sequence. If this sequence can promote amyloid formation, Sup35 protein can be turned into a phenotypically detectable prion. While this approach has been and is continued to be used successfully for some mammalian proteins associated with amyloid diseases (for review, see [30,251]), it also has certain limitations. First, some fusions antagonize Sup35 function by mechanisms not related to amyloid formation; second, in some cases when partial inactivation of Sup35 occurs due to instant amyloid formation by a chimeric protein in yeast, this system becomes difficult to apply when studying transitions between non-amyloid and amyloid states.

Another approach, specifically targeting de novo amyloid nucleation in yeast [252], has been developed on the basis of the ability of yeast prions to promote formation of other prions [253,254,255]. This approach employs the fusion of a candidate protein or protein domain to Sup35N or NM fragment that is expressed in a yeast cell separately from the full-length Sup35 protein. Transient overproduction of Sup35 or its PrD-containing fragments is known to nucleate formation of the [*PSI*^+^] prion in yeast cells [256,257]. However, this process is efficient only in the presence of another preexisting prion, such as [*PIN*^+^], a prion form of Rnq1 protein [253,254,255]. Possibly Rnq1 prion (or another prion, typically with a QN-rich domain) is needed to cross-seed the initial nucleation of the Sup35 prion in trans. However, when Sup35N or Sup35NM fragment is fused to another amyloidogenic protein (not necessarily QN-rich) in cis, such a construct can nucleate a prion on its own when overproduced (and for some highly amyloidogenic proteins, even at moderate levels of expression) [252]. The most likely scenario is that the amyloidogenic protein or domain attached to Sup35 PrD forms an amyloid aggregate in yeast, thus bringing together the Sup35 PrD regions and facilitating promoting conversion of these regions into a cross-β nucleus. Complete Sup35 protein, present in the same cell, is then immobilized into such a nucleus via the PrD-PrD interaction, and converted into a prion form, thus producing the [*PSI^+^*] prion and allowing for a phenotypic detection.

This approach has worked successfully for known mammalian amyloidogenic proteins including mouse PrP (an agent of transmissible spongiform encephalopathies, or prion diseases), Aβ (associated with Alzheimer’s disease), α-synuclein (associated with Parkinson’s disease), and amylin, or IAPP (associated with type II diabetes) [252]. Moreover, sequence alterations in PrP and Aβ that are known to antagonize prion propagation or amyloid formation also decreased the ability of respective constructs to nucleate the [*PSI*^+^] prion in yeast model system, while to the sequence alterations associated with a heritable form of the disease promoted [*PSI*^+^] nucleation. The formation of detergent-resistant aggregates by chimeric proteins and immobilization of full-length Sup35 into an aggregated state has also been confirmed by biochemical approaches [252]. In contrast, non-amyloid proteins, including those known to form globular multimeric assemblies, failed to nucleate the [*PSI*^+^] prion in the absence of pre-existing prions when fused to Sup35 PrD. Even though further studies are needed to determine if some proteins forming more complex non-globular non-amyloid assemblies, such as liquid droplets, hydrogels, or hydrophobic agglomerates, are capable of nucleating prion in yeast, existing data already establish a fusion of the protein or domain of interest to Sup35 PrD as an assay for the initial phenotypic detection of amyloidogenic properties of the proteins of various origins in yeast.

This assay is amenable to the large-scale screening and has been applied to studying human-derived library and several human proteins whose amyloidogenicity has been predicted by a computational algorithm (A. Zelinsky, N. Romanova, D. Kachkin, A. Aksenova, A. Rubel, and Y. Chernoff, unpublished data). Several new amyloidogenic human proteins have indeed been identified and are currently under detailed investigation by using standard amyloid detection techniques and studies of aggregation properties in the native environment (human cells). Some of these proteins may represent candidates for new functional amyloids in humans.

## 4. Conclusions

A number of examples of mammalian functional amyloids are described to date (see Table 1). Recently developed unbiased approaches for amyloid detection can expand our knowledge of mammalian amyloidomes very quickly. While functions of amyloids vary between different proteins and tissues, some common features could be recognized as well. Typically, in contrast to pathogenic or heritable amyloids, functional amyloids are dynamic formations that undergo structural changes in response to physiological or external signals. In response to such signals, amyloidogenic proteins can be assembled into oligomers or polymers (CPEB), solubilized from polymers into monomers (MBP-1, peptide hormones) or undergo changes in structural and functional properties (ZP proteins). For some mammalian functional amyloids, such dynamic transitions could be promoted by covalent posttranslational modifications, such as SUMOylation/ubiquitination or site-specific proteolysis. Such modifications of proteins regulating amyloid assembly or disassembly are also described for other taxa [258,259,260].

Notably, some pathogenic amyloids have been hypothesized to also play biologically positive roles. For example, Aβ (related to AD) and PrP (associated with transmissible spongiform encephalopathies) were suggested to participate in the antimicrobial defense in brains [261,262]. This indicates that some amyloid-related pathologies could represent a by-product of the functional manifestations of certain amyloids. Mechanisms of pathological amyloidoses are still poorly understood, therefore the study of functional amyloids could provide additional insights into the processes leading to amyloid pathogenicity. While functional amyloids are formed (or disassembled) in a controlled manner for performing certain biological functions, pathogenic amyloids escape the cellular or organismal control, essentially becoming the spreading disease agents. Nevertheless, molecular mechanisms responsible for the conversion of proteins to the amyloid form or for the disassembly/clearance of amyloids could be similar for functional and pathological entities [263]. Thus, further investigation of functional amyloids may help us to elaborate on new approaches to the treatment of amyloidosis.

## Figures and Tables

**Table 1:** Functional mammalian amyloids considered in this review.

Protein	Proposed Function in the Amyloid Form	Evidence for Amyloid Formation	References
Peptide hormones	Storage of hormones and pro-hormones	In vitro: EM; Luminescent conjugated polyelectrolyte probes; CD spectrum; ThT and CR staining; X-ray fiber diffraction. In vivo: CD spectrum; ThT, ThS, and CR staining with birefringence; binding to amyloid-specific antibody; X-ray fiber diffraction.	[40]
PMEL17	Templates the synthesis of melanin in melanosomes	In vitro: EM; CD spectrum; X-ray fiber diffraction; ThT and CR staining, In vivo: ThS and CR staining; resistance to detergents.	[60,87]
CPEB3	Modulation of long-term memory, synaptic plasticity	In vitro: ThS staining; CR staining with birefringence; resistance to detergents. In vivo: ThS staining; resistance to detergents. Heterologous model systems: yeast.	[99]
FXR1	Regulation of RNA stability and translation	In vitro: EM; CR staining with birefringence. In vivo: ThT, ThS and CR staining; resistance to detergents. Heterologous model systems: E. coli (C-DAG).	[120]
Zona pellucida proteins	Oocyte protection and facilitation of fertilization.	In vitro: EM; ThS staining; CR staining with birefringence. In vivo: X-ray fiber diffraction; resistance to detergents; binding to amyloid-specific antibodies; Protein aggregation disease (PAD) ligand pulldown.	[127]
RIP1 and RIP3	Regulation of necroptosis	In vitro: CD spectrum; solid-state NMR spectrum; X-ray fiber diffraction; ThT and CR staining. In vivo: ThT staining.	[147]
MBP-1	Storage of toxic antibacterial protein	In vitro: EM; X-ray fiber diffraction; ThT staining; conjugation to luminescent polyelectrolyte probes. In vivo: CR staining with birefringence; binding to amyloid-specific antibody.	[159]
CRES	Spermatozoa maturation	In vitro: EM; X-ray fiber diffraction; ThT staining; binding to amyloid specific antibody. In vivo: ThS staining; PAD ligand pulldown.	[169]
Sperm acrosomal matrix (AM)	The acrosome reaction during fertilization of oocytes.	In vivo: EM; X-ray fiber diffraction; ThS staining; binding to amyloid-specific antibodies.	[173]
PAP	Protective network in vaginal pathways	In vitro: CD spectrum; ThT staining; CR staining with birefringence; X-ray diffraction; atomic force microscopy; hydrogen-deuterium exchange; deep ultraviolet Raman resonance spectra.	[175]
MAVS	Downstream signaling in anti-viral pathways	In vitro: EM; resistance to detergents; protease resistance.	[190]
Fibrin	Component of blood cloth	In vitro: CD spectrum; ThT and CR staining; X-ray fiber diffraction.	[197]
TIA-1	Formation of stress granules	In vivo: EM; resistance to detergents. Heterologous model systems: yeast	[218]

## Abbreviations

aa	Amino acid
ACTH	Adrenocorticotropic hormone
AD	Alzheimer’s disease
AGO2	Argonaute 2 complex
AM	Acrosomal matrix
AMP	Adenine mononucleotidephosphate
AP	Amyloidogenic protein
ARE	AU-rich elements
Aβ	Amyloid beta
C-DAG	Curli-dependent amyloid generator
CARD	Caspase recruitment domains
cKO	Conditional knockout
CPEB	Cytoplasmic polyadenylation element binding protein
CD	Circular dichroism
CR	Congo red
CRH	Corticotropin releasing hormone
CTD	C-terminal domain
DD	Death domain
E-LTP	Early phase of long-term potentiation
EM	Electron microscopy
ER	Endoplasmic reticulum,
FXR	Fragile X-related protein
GAG	Glycosaminoglycans
GH	Growth hormone
GRF	Growth-hormone releasing factor
hIAPP	Human islet amyloid polypeptide
IgG	Immunoglobulin G
Igl	Immunoglobulin light chains
KD	Kinase domain
KLD	Kringle-like domain
LCP	Luminescent conjugated polyelectrolyte probes
L-LTP	Late phase of long-term potentiation
LPS	Lipopolysaccharide
MAVS	Mitochondrial antiviral signaling protein
MBP-1	Eosinophil major basic protein 1
MDA5	Melanoma differentiation-associated 5
MLKL	Mixed lineage kinase domain-like protein
MTT	3-(4,5-dimethylthiazol-2-yl)-2,5-diphenyl tetrazolium bromideformazan
NAP	Non-amyloidogenic protein
NF-κB	Nuclear factor kappa B
NMR	Nuclear magnetic resonance
NTR	N-terminal repeats
PAD	Protein aggregation disease
PAP	Prostate acidic phosphatase
PD	Parkinson’s disease
PE	Preeclampsia
PKD	Polycystic kidney disease-like domain
PrD	Prionogenic domain
PrDL	Prion-like domain
PSIA	Proteomic Screening for Identification of Amyloid proteins
Q-rich	Glutamine-rich
QN-rich	Glutamine/Asparagine-rich
RHIM	RIP homotypic interaction motifs
RNP	Ribonucleoprotein
RPT	Repeat domain
SDS	Sodium dodecyl sulfate
SGs	Stress granules
StG	Storage granule
T2D	Type 2 diabetes
ThS	Thioflavin-S
ThT	Thioflavin-T
TIA-1	T-cell intracellular antigen-1
TM	Targeting domain
TNF	Tumor necrosis factor
tPA	Tissue plasminogen activator
TSE	Transmissible spongiform encephalopathy
UTR	Untranslated terminal repeats
UV	Ultraviolet
ZP	Zona Pellucida
